# Letter to the Société de Medicine at Paris, on the Plica Polonica

**Published:** 1810-03

**Authors:** M. de La Fontaine

**Affiliations:** Surgeon en Chef to the Polish Army, and Physician to the King of Poland


### LETTER to the *Société de Medicine at Paris*, on *the Plica Polonica*. By M. de La Fontaine, Surgeon en Chef to the Polish Army, and Physician to the King of Poland.

( From the Journal de Medicine. )

I Have read in several Journals an extract from a Memoir of M. Roussille Chamseru on Plica. This memoir contains errors which it is easy to refute, by experiments founded on incontestible facts. The author seems to have seen nothing but false plica, and all his assertions are erroneous. I am prepared to answer them in a more detailed manner; in the mean time I beg leave to submit some reflections to your judgment.

I resided in Poland for 30 years. It was at Cracow that I commenced my medical career, and practiced there five years, when the late King of Poland called me into his service. While in this situation, I conceived the idea of studying the plicous disease, endemic in Poland. With this view I took several natives who were affected with plica into my own house. Having them thus under my eyes, and having an opportunity of conversing with them frequently during the day, I thought I could better trace the progress of the disease, and make some fundamental observations. I added theory to practice; I procured various works on the plica; read all the authors who had written on the subject, and who exceeded seven hundred in number; among these are some of the most distinguished characters of the last three centuries.

At Warsaw I continued my laborious inquiries; a very extensive practice in the most populous city in Poland, the hospital of the Court, and various other hospitals, afforded me ample opportunities of daily observing cases of plica, and watching its various shades, distinguishing the true from the false plica, and, above all, of minutely examining the affections, which, like masked diseases, are the precursors of plica.

After a study of fifteen years, and the treatment of several thousands of persons affected with plica, I ventured to print my work. Since that period I have never missed any opportunity of acquiring additional knowledge of this singular disease. The circumstance of the hair, in plicous cases, becoming grey, as if from the effects of age, particularly attracted my attention, not having witnessed such a case before, as well as the different heads of chronic nic diseases, which happen before or after plica, and upon which I had only noticed the most prominent features.

The difficult art of ascertaining diseases, and curing them methodically, requires long continued studies, and a series of well regulated observations, particularly when so complicated an endemic as the plica polonica is under examination. To attain this object, a long residence in the country is requisite, besides consulting and profiting by the experience of those who have preceded us; setting out on a sound theory, we ought always to have observed a prodigious number of similar cases before the crisis; for without being acquainted with the country, the inhabitants, their ways of living, manners, customs, external and internal causes which injure the body, food, air, climate, &c. without frequent intercourse with enlightened men acquainted with the subject; in short, without studying by himself this singular disease, an author cannot give any real useful precept for the treatment that ought to be applied. From what M. Roussille Chamseru asserts in his works, I maintain that he has not seen a single patient labouring under a true plica: for we cannot regard as such, the man and the dog of Posen, since, if they ever had the true plica, they were by that time free from that disease. He has not seen any thing therefore but the termination of the plica, i. e. the crisis: at this period the plica is finished; the disease has ceased, and the patient is convalescent.

The means proposed by M. Chamseru for destroying plica, are prompt and easy; and his prejudices induced him to regard them as demonstrated. He asserts, that we may cut off the plica without any danger; it is fortunate for those who are attacked with this disease, that an advice so prejudicial, thrown out at random, has met with little approbation in Poland.

If M. Chamseru had ever an opportunity of seeing patients in whom the plica was not yet declared, or in whom the plica was quite recent, and still firmly attached to the head; if he had seen this plica on the banks of the Vistula, the Narva, the Niemen, the Bug, and the Wolga, instead of observing it at Posen, which is nearer Germany; if he had seen a true plica cut, when recently formed; if he had been present at the frightful, or even mortal scenes which succeed this operation, he certainly would not have hazarded such an opinion.

The author of the memoir on plica regards want of cleanliness, the use of furred bonnets, grease, and the neglect to to comb the hair, as the sole causes of this disease. He says also, that men are more frequently attacked than women. For my part, I, who have seen and cured this disease for thirty years, maintain the contrary, and my assertion is founded on several thousand different experiments.

Women in general have more hair than men, and consequently the plicous matter in them is more easily propagated; besides, the men in Poland have their heads shaven, and are therefore less subject to be infected with plica than the women.

As to want of cleanliness, considered as a cause of plica, we shall venture to say, that the lower classes in all countries are negligent of their persons, and yet we do not find any instances of plica in other nations, as we do among the Poles.

There are hairy parts of the human body which it is not usual to comb, and yet this neglect is attended with no inconvenience. In Poland, on the contrary, the plica is frequently found in those parts, nay even children are born with plica, a circumstance which, of course, cannot proceed from want of cleanliness. It would seem also that the author has not seen the nails of the hands and toes affected with plica, since he does not speak of this circumstance in his Memoir.

Daily experience demonstrates, that no age and no condition is exempt from this disease, and even the utmost attention to cleanliness does not always preserve from infection; this proves that the plica is really a disease peculiar to Poland. All authors, however, who have hitherto written on the subject, have carefully distinguished between true and false plica. These two diseases, in fact, have very different characters, which have entirely escaped M. Chamseru. The false plica arising only from the hair being twisted, and plaited by grease and filth. The use of the comb will necessarily excite pain, and yet it is possible to use it. This kind of plica is always far removed from the head, an advantage which it possesses over the true plica, which on the contrary always renders the hair firmly adherent to the head, forming as it were one mass; the slightest touch gives the patient the most acute pain, and even frequently produces convulsions; it would therefore be very dangerons to cut a plica of this description.

None of the authors who have written on this disease, have doubted its existence, but they are not agreed with respect to its origin, the manner of treating it, and particularly as to the danger of cutting it off. Several maintain that that this operation may be performed, while others are of a contrary opinion; both ways of thinking are correct, although at first sight, they seem formally to contradict each other.

Those authors who have forbid us to touch the plica, far less to cut it, have made their observations on true plica, recently formed and strongly attached to the head: hence proceeds their erroneous opinion, that plica must in no case be cut. Others, on the contrary, have made experiments on false, or upon true plica, which have become detached from the head, and have affirmed, that it may be cut without danger; upon these two different observations have the opinion of authors been founded. I flatter myself that I have been the first to clear up the doubts, and to resolve the various questions which have arisen on this subject; because my multiplied observations have taught me that we cannot cut a true plica without great danger, when in its first stage, and adhering to the head. All the symptoms must have entirely ceased, the plica must be removed from the head at least two or three inches, and we must have seen new and sound hairs springing up which push it downwards. Lastly, the patient must have gone through a complete crisis before we can cut the true plica, whereas the false, as it proceeds from want of cleanliness only, may be cut at any time without danger.

The first partition of Poland furnished some remarkable instances of what I now advance. Among the Polish recruits raised by the new possessors, there were many young persons affected with plica. Russia, where this disease is endemic as well as in Poland, and particularly in the provinces adjoining the latter country, did not enrol any, but Austria and Prussia did. The German surgeons of that period had false notions respecting plica; the heads of the new soldiers were shaved, which caused convulsions and death in some; while others, who had undergone a similar operation, got well. New opinions continued to be started among medical men, until the publication of my work, in which the different cases, founded on authentic observations, were analysed. I pointed out the circumstances in which it was possible to cut plica with advantage. One of the best surgeons in Germany, M. Theden, sent me the following letter on the subject, (1792.) "I have read your work on plica with great pleasure: the contents will be highly useful to the Prussian army. Hitherto our Polish recruits have furnished us with sad examples of the ignorance in which we were on the subject of this disease.

Last

Last year, three young men died suddenly from epilepsy, when we cut off the plica from them; this will not happen in future, for we shall strictly adhere to your directions. I have made an application to the Prussian Government, with a view to procure a copy of your work for every regimental surgeon, and have requested, that these gentlemen may be instructed to adopt your practice in future."

The terrors evinced by the Poles themselves, when it is proposed to cut the plica, does not proceed, as M. Chamseru pretends, from national prejudices, but from many examples of dreadful accidents, occasioned by the imprudence which they have sometimes witnessed of cutting true plica when strongly attached to the head; hence, the Poles refused to submit to the operation, whatever may be the kind of plica they labour under, and we frequently see true plica entirely detached from the head, which remain until they fall of themselves, or until a new plica is formed. There have been instances of double or even triple plica. M. Jenker, teacher of Surgery at Berlin, has in his possession, a true plica eleven ells in length: plica of this description are surely unknown to M. Chamseru.

I have seen an instance of a man attacked with a pleurisy, whose pains ceased the moment that his hair (which was perfectly sound two hours before) became truly plicous. I have also seen apoplexies, convulsions, and epilepsies disappear immediately on the plica being formed as a crisis. In my large work, I enter upon all these causes at greater length. I have observed phenomena of a most extraordinary kind in the operation for cataract; *i. e.* in cases where the liquor of Morgagni was opaque, the crystaline remained perfectly clear and transparent in plicous patients. Since the appearance of my work on plica, I have undertaken fewer hazardous operations in such patients, and on this account, I have had the good fortune to restore to sight several blind persons, without any other treatment, and without the most trifling operation. It is proved beyond doubt, that in no country in the world, are there so many blind persons cured as in Poland, particularly those who are attacked with amaurosis, the most formidable of all the diseases of the eye: I do not allude to the ordinary diseases of that organ.

I have confined myself to the foregoing examples, because I think them sufficient to fulfil the object I had in view, namely, that of refuting the assertions of M. Chamseru. The cause of humanity, and above all, the inhabitants of the North of Cracow would be considerable gainers, gainers, if as this author alleges, the plica polonica was not a disease; but it is very difficult to retain the opinion of more than 700 authors; and it is still more difficult to extirpate an endemic disease, by hazarding a theory which cannot be supported by positive facts.

